# Bis(1-ammonio­ethane-1,1-diyl­diphos­phonato-κ^2^ *O*,*O*′)diaqua­cobalt(II) nona­hydrate

**DOI:** 10.1107/S1600536810013681

**Published:** 2010-04-17

**Authors:** Vladimir V. Bon, Anatolij V. Dudko, Alexandra N. Kozachkova, Vasily I. Pekhnyo, Natalia V. Tsaryk

**Affiliations:** a Institute of General and Inorganic Chemistry, NAS Ukraine, Kyiv, prosp. Palladina 32/34, 03680, Ukraine

## Abstract

In the title compound, [Co(C_2_H_8_NO_6_P_2_)_2_(H_2_O)_2_]·9H_2_O, the Co^II^ atom has a slightly distorted octa­hedral coordination environment consisting of four deprotonated phospho­nate O atoms of two independent 1-amino­ethyl­idendiphospho­nate anions and complemented by the O atoms of two water mol­ecules in *cis* positions. The anions exists in the zwitterionic form (protonated amino group and two deprotonated phospho­nate O atoms) and constitute two six-membered chelate rings. The crystal structure also contains nine partly disordered uncoordinated water mol­ecules, which create an extensive three-dimensional network of strong O—H⋯O and N—H⋯O hydrogen bonds.

## Related literature

For general background to organic diphospho­nic acids, see: Matczak-Jon & Videnova-Adrabinska (2005 ▶). For applications of transition metal bis­phospho­nates, see: Eberhardt *et al.* (2005 ▶). For related structures, see: Xiang *et al.* (2007 ▶); Yin *et al.* (2005 ▶); Dudko *et al.* (2009 ▶).
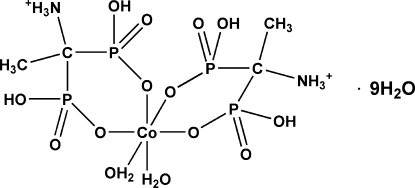


## Experimental

### 

#### Crystal data

[Co(C_2_H_8_NO_6_P_2_)_2_(H_2_O)_2_]·9H_2_O
*M* *_r_* = 665.17
Monoclinic, 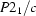

*a* = 15.1925 (3) Å
*b* = 13.2046 (2) Å
*c* = 12.9688 (2) Å
β = 106.0866 (11)°
*V* = 2499.81 (7) Å^3^
*Z* = 4
Mo *K*α radiation
μ = 1.04 mm^−1^
*T* = 173 K
0.30 × 0.24 × 0.20 mm

#### Data collection

Bruker APEX-II CCD diffractometer
Absorption correction: multi-scan (*SADABS*; Bruker, 2005 ▶) *T*_min_ = 0.749, *T*_max_ = 0.820
51914 measured reflections
6273 independent reflections
5626 reflections with *I* > 2σ(*I*)
*R*_int_ = 0.030

#### Refinement

*R*[*F* ^2^ > 2σ(*F* ^2^)] = 0.032
*wR*(*F* ^2^) = 0.090
*S* = 1.12
6273 reflections
401 parameters
H atoms treated by a mixture of independent and constrained refinement
Δρ_max_ = 0.73 e Å^−3^
Δρ_min_ = −0.29 e Å^−3^

### 

Data collection: *APEX2* (Bruker, 2005 ▶); cell refinement: *SAINT* (Bruker, 2005 ▶); data reduction: *SAINT*; program(s) used to solve structure: *SHELXS97* (Sheldrick, 2008 ▶); program(s) used to refine structure: *SHELXL97* (Sheldrick, 2008 ▶); molecular graphics: *SHELXTL* (Sheldrick, 2008 ▶); software used to prepare material for publication: *publCIF* (Westrip, 2009 ▶).

## Supplementary Material

Crystal structure: contains datablocks I, global. DOI: 10.1107/S1600536810013681/wm2322sup1.cif

Structure factors: contains datablocks I. DOI: 10.1107/S1600536810013681/wm2322Isup2.hkl

Additional supplementary materials:  crystallographic information; 3D view; checkCIF report

## Figures and Tables

**Table 1:** Selected bond lengths (Å)

Co1—O7	2.0697 (14)
Co1—O13	2.0747 (17)
Co1—O10	2.0771 (15)
Co1—O14	2.0837 (16)
Co1—O1	2.1007 (15)
Co1—O4	2.1201 (15)

**Table 2:** Hydrogen-bond geometry (Å, °)

*D*—H⋯*A*	*D*—H	H⋯*A*	*D*⋯*A*	*D*—H⋯*A*
N1—H11*N*⋯O17	0.86 (3)	2.00 (3)	2.836 (3)	165 (3)
N1—H12*N*⋯O7	0.81 (3)	1.98 (3)	2.785 (2)	175 (3)
N1—H13*N*⋯O15^i^	0.85 (3)	1.98 (3)	2.810 (2)	165 (3)
N2—H21*N*⋯O23*A*	0.91 (3)	1.94 (3)	2.808 (4)	159 (3)
N2—H22*N*⋯O4	0.90 (3)	2.05 (3)	2.944 (2)	173 (3)
N2—H23*N*⋯O16	0.90 (3)	1.90 (3)	2.783 (3)	164 (3)
O3—H3*O*⋯O6^ii^	0.66 (3)	1.92 (3)	2.570 (2)	169 (4)
O5—H5*O*⋯O2^i^	0.76 (3)	1.84 (3)	2.592 (2)	170 (3)
O8—H8*O*⋯O11^iii^	0.70 (3)	1.81 (3)	2.508 (2)	169 (3)
O12—H12*O*⋯O9^iv^	0.83 (3)	1.71 (3)	2.517 (2)	165 (3)
O13—H131⋯O6^ii^	0.79 (3)	1.92 (3)	2.691 (2)	166 (3)
O13—H132⋯O20*A*	0.77 (3)	1.92 (3)	2.671 (3)	165 (3)
O14—H141⋯O18*A*^v^	0.82 (3)	1.91 (3)	2.697 (3)	163 (3)
O14—H142⋯O9^iv^	0.74 (3)	1.95 (3)	2.688 (2)	172 (3)
O15—H151⋯O1	0.80 (3)	2.01 (3)	2.803 (2)	177 (3)
O15—H152⋯O14	0.66 (3)	2.50 (3)	2.946 (2)	127 (3)
O15—H152⋯O12^iv^	0.66 (3)	2.54 (3)	3.049 (2)	136 (3)
O16—H161⋯O15^i^	0.85 (4)	1.94 (4)	2.788 (3)	176 (3)
O16—H162⋯O17^i^	0.89 (4)	1.97 (4)	2.844 (3)	170 (3)
O17—H171⋯O21^vi^	0.75 (3)	2.14 (4)	2.795 (3)	147 (3)
O17—H172⋯O18*A*^vii^	0.73 (4)	2.13 (4)	2.845 (3)	167 (4)
O19—H191⋯O13	0.84 (4)	2.27 (4)	3.063 (3)	158 (3)
O19—H192⋯O2^v^	0.75 (4)	2.04 (4)	2.773 (3)	166 (4)
O21—H211⋯O19	0.80 (4)	2.05 (4)	2.815 (3)	162 (4)
O21—H212⋯O19^viii^	1.00 (4)	1.93 (4)	2.914 (3)	169 (3)

Symmetry codes: (i) 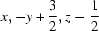; (ii) 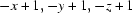; (iii) 
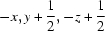
; (iv) 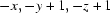; (v) 
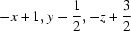
; (vi) 
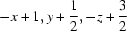
; (vii) 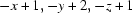; (viii) 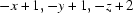.

**Fig. 1.**
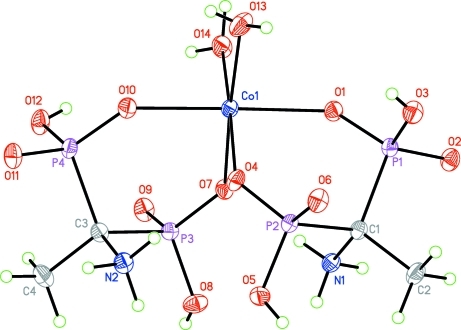
The molecular structure of title compound showing 50% probability displacement ellipsoids for the non-hydrogen atoms. Solvent water molecules are omitted for clarity.

**Fig. 2.**
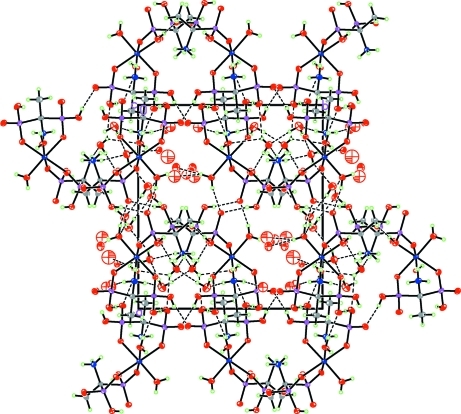
Crystal packing of title compound, in a projection along the c axis. Dashed lines indicate hydrogen bonds.

## supplementary crystallographic information

### Comment

Organic diphosphonic acids are potentially very powerful chelating agents used in metal extractions and are tested by the pharmaceutical industry for use as efficient drugs preventing calcification and inhibiting bone resorption (Matczak-Jon *et al.*, 2005). There is evidence that application of transition metal bisphosphonates can improve fixation of cementless metal implants by enhancing the extent of osseointegration (Eberhardt *et al.*, 2005). In this respect, a detailed structure-correlated study of the individual properties and the complex-forming driving factors is desired in order to sufficiently understand bisphosphonate physiological activities.

Several structures of Co^II^ aminoethylidenediphosphonates have been reported previously (Xiang *et al.* 2007; Yin *et al.* 2005). The main difference between these structures and the title compound is the presence of two water molecules instead of a 1,10-phenanthroline ligand in the coordination environment of the transition metal ion (Xiang *et al.* 2007), leading also to a different symmetry.

The asymmetric unit of the title compound contains one molecule of the complex (Fig.1). Two 1-aminoethylidendiphosphonate anions chelate the central metal ion via two oxygen atoms from phosphonate groups forming six-membered non-planar metalla rings. Two water molecules complement the slightly distorted octahedral coordination environment of Co in *cis-*position. The Co—O bond lengths have expected values and conform with the previously reported related structures (Xiang *et al.*, 2007). The values of the O—Co—O angles are in the range from 89.23 (7)° to 91.54 (5)°. The Co1—O1—P1—C1—P2—O4 and Co1—O7—P3—C3—P4—O10 metalla cycles have an envelope conformation with the C1 and C3 atoms out of plane by 0.850 (2) Å and 0.795 (2) Å, respectively. The dihedral angle between the planar fragments Co1—O1—P1—P2—O4 and Co1—O7—P3—P4—O10 is 84.20 (3)°. The coordinated ligand molecules exists in the zwitterionic form with a proton transfer from one of the phosphonic groups to the amino group which is representative for all 1-aminodiphosphonic acids. In addition, the amino group does also not participate in coordination (Dudko *et al.* 2009).

In the crystal structure of the title compound, nine solvent water molecules are present. Such an amount of solvent molecules could be explained by the presence of two coordinated water molecules in addition to the more hydrophilic phosphonate groups. As a result, a 3-D network of mostly strong O—H···O and N—H···O hydrogen bonds is observed in the structure (Fig. 2; Table 1). Several H-bonds can not be unambiguously derived from the model because some of the crystal lattice water molecules are disordered.

### Experimental

Light pink crystals of the title compound were obtained from the mixture of 10 ml (10 ^-2^ mol/l) water solution of Co(NO_3_)_2_ with 20 ml (10 ^-2^ mol/l) solution of 1-aminoethylidendiphosphonic acid. The combined solution was stored in a dark place for slow evaporation. After 20 days of staying, suitable crystals for X-ray data collection were obtained.

### Refinement

In the crystal structure of the title compound, O atoms O18 and O20 are disordered over two sites with occupancies 0.87/0.13. Disordered O atoms O22 and O23 were treated with occupancies 0.88/0.12 and 0.71/0.29, respectively. The major component of the disordered site was refined anisotropically, the corresponding minor occupied sites were refined isotropically. Hydrogen atoms bonded to the disordered oxygen atoms could not be located from difference Fourier maps and were eventually omitted from refinement. Other H atoms bonded to N and O atoms were located in a difference map and refined freely with Uiso(H) = 1.5Ueq(N) and Uiso(H) = 1.2Ueq(O), respectively. Methyl hydrogens atoms were positioned geometrically and were refined using a riding model with C—H = 0.98 Å for CH_3_ [Uiso(H) = 1.5Ueq(C)].

### Crystal data

**Table:**

[Co(C_2_H_8_NO_6_P_2_)_2_(H_2_O)_2_]·9H_2_O	*F*(000) = 1388
*M**_r_* = 665.17	*D*_x_ = 1.767 Mg m^−^^3^
Monoclinic, *P*2_1_/*c*	Mo *K*α radiation, λ = 0.71073 Å
Hall symbol: -P 2ybc	Cell parameters from 9983 reflections
*a* = 15.1925 (3) Å	θ = 2.3–28.4°
*b* = 13.2046 (2) Å	µ = 1.04 mm^−^^1^
*c* = 12.9688 (2) Å	*T* = 173 K
β = 106.0866 (11)°	Block, light pink
*V* = 2499.81 (7) Å^3^	0.30 × 0.24 × 0.20 mm
*Z* = 4	

### Data collection

**Table:**

Bruker APEX-II CCD diffractometer	6273 independent reflections
Radiation source: fine-focus sealed tube	5626 reflections with *I* > 2σ(*I*)
graphite	*R*_int_ = 0.030
Detector resolution: 8.33 pixels mm^-1^	θ_max_ = 28.4°, θ_min_ = 1.4°
φ and ω scans	*h* = −19→20
Absorption correction: multi-scan (*SADABS*; Bruker, 2005)	*k* = −17→17
*T*_min_ = 0.749, *T*_max_ = 0.820	*l* = −17→17
51914 measured reflections	

### Refinement

**Table:**

Refinement on *F*^2^	Primary atom site location: structure-invariant direct methods
Least-squares matrix: full	Secondary atom site location: difference Fourier map
*R*[*F*^2^ > 2σ(*F*^2^)] = 0.032	Hydrogen site location: inferred from neighbouring sites
*wR*(*F*^2^) = 0.090	H atoms treated by a mixture of independent and constrained refinement
*S* = 1.12	*w* = 1/[σ^2^(*F*_o_^2^) + (0.0396*P*)^2^ + 3.3375*P*] where *P* = (*F*_o_^2^ + 2*F*_c_^2^)/3
6273 reflections	(Δ/σ)_max_ = 0.001
401 parameters	Δρ_max_ = 0.73 e Å^−^^3^
0 restraints	Δρ_min_ = −0.29 e Å^−^^3^

### Special details

**Table:**

Geometry. All esds (except the esd in the dihedral angle between two l.s. planes) are estimated using the full covariance matrix. The cell esds are taken into account individually in the estimation of esds in distances, angles and torsion angles; correlations between esds in cell parameters are only used when they are defined by crystal symmetry. An approximate (isotropic) treatment of cell esds is used for estimating esds involving l.s. planes.
Refinement. Refinement of *F*^2^ against ALL reflections. The weighted *R*-factor wR and goodness of fit *S* are based on *F*^2^, conventional *R*-factors *R* are based on *F*, with *F* set to zero for negative *F*^2^. The threshold expression of *F*^2^ > σ(*F*^2^) is used only for calculating *R*-factors(gt) etc. and is not relevant to the choice of reflections for refinement. *R*-factors based on *F*^2^ are statistically about twice as large as those based on *F*, and *R*- factors based on ALL data will be even larger.

### Fractional atomic coordinates and isotropic or equivalent isotropic displacement parameters (Å^2^)

**Table:**

	*x*	*y*	*z*	*U*_iso_*/*U*_eq_	Occ. (<1)
Co1	0.255576 (18)	0.49258 (2)	0.52538 (2)	0.01238 (8)	
P1	0.40125 (3)	0.68696 (4)	0.57579 (4)	0.01281 (11)	
P2	0.37271 (3)	0.58209 (4)	0.35868 (4)	0.01196 (11)	
P3	0.06525 (3)	0.58713 (4)	0.36181 (4)	0.01226 (11)	
P4	0.08900 (3)	0.35638 (4)	0.36325 (4)	0.01415 (11)	
C1	0.36957 (13)	0.70306 (15)	0.42887 (16)	0.0131 (4)	
C2	0.43053 (15)	0.78295 (17)	0.39660 (17)	0.0188 (4)	
H21C	0.4116	0.7914	0.3185	0.028*	
H22C	0.4946	0.7608	0.4198	0.028*	
H23C	0.4243	0.8477	0.4309	0.028*	
C3	0.06352 (13)	0.47197 (16)	0.28100 (16)	0.0141 (4)	
C4	−0.02844 (14)	0.46254 (19)	0.19417 (17)	0.0204 (4)	
H41C	−0.0374	0.5219	0.1470	0.031*	
H42C	−0.0784	0.4586	0.2283	0.031*	
H43C	−0.0282	0.4011	0.1518	0.031*	
N1	0.27212 (12)	0.74065 (14)	0.39596 (15)	0.0142 (3)	
H11N	0.2720 (19)	0.799 (2)	0.426 (2)	0.021*	
H12N	0.237 (2)	0.701 (2)	0.412 (2)	0.021*	
H13N	0.2560 (19)	0.749 (2)	0.328 (2)	0.021*	
N2	0.13796 (12)	0.48190 (15)	0.22493 (15)	0.0165 (3)	
H21N	0.1347 (19)	0.429 (2)	0.179 (2)	0.025*	
H22N	0.193 (2)	0.486 (2)	0.273 (2)	0.025*	
H23N	0.128 (2)	0.538 (2)	0.183 (2)	0.025*	
O1	0.33239 (10)	0.61709 (12)	0.60220 (11)	0.0160 (3)	
O2	0.40782 (10)	0.79109 (11)	0.62352 (12)	0.0181 (3)	
O3	0.49936 (10)	0.64009 (13)	0.60579 (13)	0.0188 (3)	
H3O	0.502 (2)	0.590 (2)	0.605 (2)	0.023*	
O4	0.31168 (10)	0.50763 (11)	0.39358 (12)	0.0154 (3)	
O5	0.32357 (10)	0.60670 (12)	0.23804 (12)	0.0171 (3)	
H5O	0.3517 (19)	0.640 (2)	0.211 (2)	0.020*	
O6	0.47085 (10)	0.55160 (12)	0.37641 (12)	0.0178 (3)	
O7	0.15729 (9)	0.59446 (11)	0.44381 (11)	0.0151 (3)	
O8	0.06089 (11)	0.67523 (12)	0.28140 (13)	0.0191 (3)	
H8O	0.017 (2)	0.695 (2)	0.258 (2)	0.023*	
O9	−0.01614 (10)	0.58374 (12)	0.40569 (12)	0.0179 (3)	
O10	0.17939 (10)	0.37086 (11)	0.44643 (12)	0.0179 (3)	
O11	0.08724 (10)	0.26901 (12)	0.28832 (12)	0.0195 (3)	
O12	0.00608 (11)	0.34512 (13)	0.41132 (13)	0.0199 (3)	
H12O	0.0100 (19)	0.378 (2)	0.467 (2)	0.024*	
O13	0.35601 (12)	0.39698 (14)	0.61576 (15)	0.0238 (4)	
H131	0.407 (2)	0.403 (2)	0.614 (2)	0.029*	
H132	0.340 (2)	0.341 (3)	0.611 (2)	0.029*	
O14	0.18902 (11)	0.48630 (13)	0.64577 (13)	0.0198 (3)	
H141	0.210 (2)	0.456 (2)	0.702 (3)	0.024*	
H142	0.140 (2)	0.472 (2)	0.630 (2)	0.024*	
O15	0.19611 (13)	0.70714 (14)	0.67947 (14)	0.0234 (4)	
H151	0.235 (2)	0.680 (2)	0.660 (2)	0.028*	
H152	0.165 (2)	0.671 (3)	0.674 (3)	0.028*	
O16	0.10482 (14)	0.62776 (16)	0.06388 (15)	0.0310 (4)	
H161	0.130 (2)	0.679 (3)	0.099 (3)	0.037*	
H162	0.143 (2)	0.610 (3)	0.026 (3)	0.037*	
O17	0.24161 (13)	0.93732 (14)	0.46692 (16)	0.0265 (4)	
H171	0.287 (2)	0.949 (3)	0.506 (3)	0.032*	
H172	0.229 (2)	0.979 (3)	0.429 (3)	0.032*	
O19	0.44927 (14)	0.42724 (16)	0.85539 (17)	0.0329 (4)	
H191	0.415 (2)	0.433 (3)	0.793 (3)	0.039*	
H192	0.483 (3)	0.385 (3)	0.866 (3)	0.039*	
O21	0.60125 (14)	0.55256 (16)	0.94457 (19)	0.0359 (4)	
H211	0.559 (3)	0.523 (3)	0.907 (3)	0.043*	
H212	0.590 (2)	0.567 (3)	1.015 (3)	0.043*	
O18A	0.77849 (14)	0.88751 (17)	0.66533 (16)	0.0234 (8)	0.873 (11)
O20A	0.3072 (2)	0.20567 (19)	0.6380 (3)	0.0415 (11)	0.869 (14)
O22A	0.67469 (18)	0.7176 (2)	0.6366 (4)	0.0409 (12)	0.876 (13)
O23A	0.1129 (3)	0.3552 (6)	0.0452 (6)	0.0420 (16)	0.71 (3)
O18B	0.747 (3)	0.840 (3)	0.668 (3)	0.101 (12)*	0.127 (11)
O20B	0.350 (3)	0.197 (2)	0.684 (3)	0.076 (10)*	0.131 (14)
O22B	0.6582 (15)	0.7397 (17)	0.579 (3)	0.042 (6)*	0.124 (13)
O23B	0.1074 (8)	0.3242 (16)	0.0717 (14)	0.048 (3)*	0.29 (3)

### Atomic displacement parameters (Å^2^)

**Table:**

	*U*^11^	*U*^22^	*U*^33^	*U*^12^	*U*^13^	*U*^23^
Co1	0.01106 (13)	0.01304 (14)	0.01351 (13)	−0.00105 (9)	0.00416 (10)	0.00019 (10)
P1	0.0123 (2)	0.0130 (2)	0.0135 (2)	−0.00120 (18)	0.00427 (18)	−0.00135 (18)
P2	0.0114 (2)	0.0119 (2)	0.0137 (2)	−0.00023 (17)	0.00541 (17)	−0.00051 (18)
P3	0.0101 (2)	0.0140 (2)	0.0133 (2)	0.00027 (17)	0.00432 (17)	0.00080 (18)
P4	0.0142 (2)	0.0133 (2)	0.0157 (2)	−0.00344 (18)	0.00540 (18)	−0.00208 (19)
C1	0.0119 (8)	0.0131 (9)	0.0154 (9)	−0.0009 (7)	0.0056 (7)	−0.0006 (7)
C2	0.0221 (10)	0.0161 (10)	0.0206 (10)	−0.0057 (8)	0.0102 (8)	−0.0005 (8)
C3	0.0121 (8)	0.0177 (10)	0.0135 (8)	−0.0022 (7)	0.0053 (7)	−0.0013 (7)
C4	0.0162 (9)	0.0272 (12)	0.0164 (9)	−0.0025 (8)	0.0020 (8)	−0.0024 (9)
N1	0.0146 (8)	0.0126 (9)	0.0157 (8)	0.0005 (6)	0.0049 (6)	0.0004 (7)
N2	0.0154 (8)	0.0186 (9)	0.0169 (8)	−0.0017 (7)	0.0064 (7)	−0.0012 (7)
O1	0.0161 (7)	0.0180 (7)	0.0150 (7)	−0.0036 (6)	0.0062 (5)	−0.0009 (6)
O2	0.0222 (7)	0.0143 (7)	0.0193 (7)	−0.0018 (6)	0.0083 (6)	−0.0045 (6)
O3	0.0143 (7)	0.0166 (7)	0.0246 (8)	0.0010 (6)	0.0038 (6)	−0.0001 (7)
O4	0.0170 (7)	0.0132 (7)	0.0189 (7)	−0.0030 (5)	0.0095 (6)	−0.0016 (5)
O5	0.0183 (7)	0.0189 (8)	0.0143 (7)	−0.0020 (6)	0.0050 (5)	0.0013 (6)
O6	0.0125 (6)	0.0175 (7)	0.0249 (7)	0.0006 (6)	0.0078 (6)	−0.0010 (6)
O7	0.0119 (6)	0.0145 (7)	0.0177 (7)	0.0005 (5)	0.0020 (5)	−0.0005 (6)
O8	0.0159 (7)	0.0200 (8)	0.0223 (8)	0.0043 (6)	0.0068 (6)	0.0073 (6)
O9	0.0135 (7)	0.0236 (8)	0.0192 (7)	−0.0022 (6)	0.0088 (6)	−0.0036 (6)
O10	0.0184 (7)	0.0147 (7)	0.0188 (7)	−0.0015 (6)	0.0024 (6)	−0.0002 (6)
O11	0.0185 (7)	0.0187 (8)	0.0221 (7)	−0.0035 (6)	0.0070 (6)	−0.0068 (6)
O12	0.0206 (7)	0.0221 (8)	0.0204 (7)	−0.0081 (6)	0.0112 (6)	−0.0056 (6)
O13	0.0156 (7)	0.0193 (8)	0.0364 (9)	−0.0003 (6)	0.0071 (7)	0.0082 (7)
O14	0.0145 (7)	0.0269 (9)	0.0186 (8)	−0.0058 (6)	0.0054 (6)	0.0001 (6)
O15	0.0258 (9)	0.0214 (9)	0.0252 (8)	−0.0049 (7)	0.0108 (7)	−0.0066 (7)
O16	0.0389 (10)	0.0333 (10)	0.0234 (8)	−0.0088 (8)	0.0127 (8)	−0.0040 (8)
O17	0.0268 (9)	0.0222 (9)	0.0283 (9)	0.0019 (7)	0.0036 (7)	−0.0002 (7)
O19	0.0286 (9)	0.0351 (11)	0.0330 (10)	0.0094 (8)	0.0052 (8)	−0.0034 (8)
O21	0.0277 (10)	0.0321 (11)	0.0470 (12)	0.0015 (8)	0.0086 (9)	−0.0061 (9)
O18A	0.0236 (11)	0.0192 (12)	0.0270 (11)	−0.0037 (8)	0.0064 (8)	−0.0031 (8)
O20A	0.0413 (19)	0.0277 (14)	0.055 (2)	−0.0005 (10)	0.0119 (15)	0.0096 (11)
O22A	0.0299 (13)	0.0307 (14)	0.064 (3)	−0.0050 (10)	0.0168 (13)	−0.0156 (15)
O23A	0.063 (2)	0.034 (3)	0.032 (2)	−0.0083 (17)	0.0194 (16)	−0.0152 (19)

### Geometric parameters (Å, °)

**Table:**

Co1—O7	2.0697 (14)	C3—C4	1.537 (3)
Co1—O13	2.0747 (17)	C4—H41C	0.9800
Co1—O10	2.0771 (15)	C4—H42C	0.9800
Co1—O14	2.0837 (16)	C4—H43C	0.9800
Co1—O1	2.1007 (15)	N1—H11N	0.86 (3)
Co1—O4	2.1201 (15)	N1—H12N	0.81 (3)
P1—O2	1.4999 (15)	N1—H13N	0.85 (3)
P1—O1	1.5039 (15)	N2—H21N	0.91 (3)
P1—O3	1.5604 (16)	N2—H22N	0.90 (3)
P1—C1	1.844 (2)	N2—H23N	0.90 (3)
P2—O6	1.4993 (15)	O3—H3O	0.66 (3)
P2—O4	1.5047 (15)	O5—H5O	0.76 (3)
P2—O5	1.5696 (15)	O8—H8O	0.70 (3)
P2—C1	1.846 (2)	O12—H12O	0.83 (3)
P3—O9	1.4982 (15)	O13—H131	0.79 (3)
P3—O7	1.5071 (14)	O13—H132	0.77 (3)
P3—O8	1.5515 (16)	O14—H141	0.82 (3)
P3—C3	1.843 (2)	O14—H142	0.74 (3)
P4—O11	1.5038 (16)	O15—H151	0.80 (3)
P4—O10	1.5050 (15)	O15—H152	0.66 (3)
P4—O12	1.5599 (16)	O16—H161	0.85 (4)
P4—C3	1.841 (2)	O16—H162	0.89 (4)
C1—N1	1.507 (3)	O17—H171	0.75 (3)
C1—C2	1.536 (3)	O17—H172	0.73 (4)
C2—H21C	0.9800	O19—H191	0.84 (4)
C2—H22C	0.9800	O19—H192	0.75 (4)
C2—H23C	0.9800	O21—H211	0.80 (4)
C3—N2	1.510 (3)	O21—H212	1.00 (4)
			
O7—Co1—O13	176.20 (7)	H21C—C2—H22C	109.5
O7—Co1—O10	91.52 (6)	C1—C2—H23C	109.5
O13—Co1—O10	91.71 (7)	H21C—C2—H23C	109.5
O7—Co1—O14	88.73 (6)	H22C—C2—H23C	109.5
O13—Co1—O14	89.20 (7)	N2—C3—C4	107.71 (16)
O10—Co1—O14	91.08 (6)	N2—C3—P4	106.60 (14)
O7—Co1—O1	87.76 (6)	C4—C3—P4	111.09 (15)
O13—Co1—O1	89.05 (7)	N2—C3—P3	107.91 (14)
O10—Co1—O1	178.83 (6)	C4—C3—P3	110.50 (15)
O14—Co1—O1	89.83 (6)	P4—C3—P3	112.77 (10)
O7—Co1—O4	85.48 (6)	C3—C4—H41C	109.5
O13—Co1—O4	96.63 (7)	C3—C4—H42C	109.5
O10—Co1—O4	88.21 (6)	H41C—C4—H42C	109.5
O14—Co1—O4	174.14 (6)	C3—C4—H43C	109.5
O1—Co1—O4	90.82 (6)	H41C—C4—H43C	109.5
O2—P1—O1	116.06 (9)	H42C—C4—H43C	109.5
O2—P1—O3	108.12 (9)	C1—N1—H11N	106.9 (18)
O1—P1—O3	112.13 (9)	C1—N1—H12N	112 (2)
O2—P1—C1	106.81 (9)	H11N—N1—H12N	112 (3)
O1—P1—C1	107.89 (9)	C1—N1—H13N	108.4 (19)
O3—P1—C1	105.14 (9)	H11N—N1—H13N	108 (3)
O6—P2—O4	116.61 (9)	H12N—N1—H13N	109 (3)
O6—P2—O5	112.75 (9)	C3—N2—H21N	109.5 (18)
O4—P2—O5	105.82 (9)	C3—N2—H22N	110.4 (19)
O6—P2—C1	108.50 (9)	H21N—N2—H22N	112 (3)
O4—P2—C1	108.35 (9)	C3—N2—H23N	110.0 (18)
O5—P2—C1	104.02 (9)	H21N—N2—H23N	106 (3)
O9—P3—O7	115.85 (9)	H22N—N2—H23N	109 (3)
O9—P3—O8	113.06 (9)	P1—O1—Co1	134.61 (9)
O7—P3—O8	106.53 (9)	P1—O3—H3O	116 (3)
O9—P3—C3	107.95 (9)	P2—O4—Co1	136.28 (9)
O7—P3—C3	108.57 (9)	P2—O5—H5O	114 (2)
O8—P3—C3	104.19 (9)	P3—O7—Co1	135.71 (9)
O11—P4—O10	114.20 (9)	P3—O8—H8O	115 (3)
O11—P4—O12	108.26 (9)	P4—O10—Co1	136.45 (10)
O10—P4—O12	113.78 (9)	P4—O12—H12O	115 (2)
O11—P4—C3	107.37 (9)	Co1—O13—H131	120 (2)
O10—P4—C3	108.28 (9)	Co1—O13—H132	112 (2)
O12—P4—C3	104.31 (9)	H131—O13—H132	112 (3)
N1—C1—C2	108.41 (17)	Co1—O14—H141	123 (2)
N1—C1—P1	106.58 (13)	Co1—O14—H142	117 (2)
C2—C1—P1	110.87 (14)	H141—O14—H142	104 (3)
N1—C1—P2	107.64 (13)	H151—O15—H152	102 (4)
C2—C1—P2	111.10 (14)	H161—O16—H162	104 (3)
P1—C1—P2	112.02 (11)	H171—O17—H172	109 (4)
C1—C2—H21C	109.5	H191—O19—H192	117 (4)
C1—C2—H22C	109.5	H211—O21—H212	110 (3)
			
O2—P1—C1—N1	−69.60 (15)	O8—P3—C3—C4	−65.37 (16)
O1—P1—C1—N1	55.84 (15)	O9—P3—C3—P4	−69.94 (12)
O3—P1—C1—N1	175.67 (13)	O7—P3—C3—P4	56.38 (12)
O2—P1—C1—C2	48.19 (16)	O8—P3—C3—P4	169.62 (10)
O1—P1—C1—C2	173.63 (14)	O2—P1—O1—Co1	152.91 (11)
O3—P1—C1—C2	−66.54 (16)	O3—P1—O1—Co1	−82.16 (14)
O2—P1—C1—P2	172.92 (9)	C1—P1—O1—Co1	33.15 (15)
O1—P1—C1—P2	−61.64 (12)	O7—Co1—O1—P1	−86.87 (13)
O3—P1—C1—P2	58.19 (12)	O13—Co1—O1—P1	95.20 (13)
O6—P2—C1—N1	171.19 (13)	O14—Co1—O1—P1	−175.60 (13)
O4—P2—C1—N1	−61.33 (15)	O4—Co1—O1—P1	−1.42 (13)
O5—P2—C1—N1	50.94 (15)	O6—P2—O4—Co1	102.36 (14)
O6—P2—C1—C2	52.64 (16)	O5—P2—O4—Co1	−131.38 (13)
O4—P2—C1—C2	−179.87 (14)	C1—P2—O4—Co1	−20.33 (16)
O5—P2—C1—C2	−67.61 (15)	O7—Co1—O4—P2	81.31 (13)
O6—P2—C1—P1	−71.96 (12)	O13—Co1—O4—P2	−95.52 (14)
O4—P2—C1—P1	55.52 (12)	O10—Co1—O4—P2	172.97 (14)
O5—P2—C1—P1	167.78 (10)	O1—Co1—O4—P2	−6.38 (14)
O11—P4—C3—N2	−60.67 (15)	O9—P3—O7—Co1	92.63 (14)
O10—P4—C3—N2	63.09 (15)	O8—P3—O7—Co1	−140.66 (12)
O12—P4—C3—N2	−175.41 (13)	C3—P3—O7—Co1	−28.97 (15)
O11—P4—C3—C4	56.41 (16)	O10—Co1—O7—P3	1.53 (13)
O10—P4—C3—C4	−179.83 (14)	O14—Co1—O7—P3	−89.51 (13)
O12—P4—C3—C4	−58.34 (16)	O1—Co1—O7—P3	−179.38 (13)
O11—P4—C3—P3	−178.91 (10)	O4—Co1—O7—P3	89.61 (13)
O10—P4—C3—P3	−55.15 (12)	O11—P4—O10—Co1	146.29 (12)
O12—P4—C3—P3	66.35 (12)	O12—P4—O10—Co1	−88.72 (15)
O9—P3—C3—N2	172.59 (13)	C3—P4—O10—Co1	26.74 (16)
O7—P3—C3—N2	−61.09 (15)	O7—Co1—O10—P4	−0.20 (14)
O8—P3—C3—N2	52.15 (15)	O13—Co1—O10—P4	177.79 (14)
O9—P3—C3—C4	55.06 (16)	O14—Co1—O10—P4	88.55 (14)
O7—P3—C3—C4	−178.61 (14)	O4—Co1—O10—P4	−85.63 (14)

### Hydrogen-bond geometry (Å, °)

**Table:**

*D*—H···*A*	*D*—H	H···*A*	*D*···*A*	*D*—H···*A*
N1—H11N···O17	0.86 (3)	2.00 (3)	2.836 (3)	165 (3)
N1—H12N···O7	0.81 (3)	1.98 (3)	2.785 (2)	175 (3)
N1—H13N···O15^i^	0.85 (3)	1.98 (3)	2.810 (2)	165 (3)
N2—H21N···O23A	0.91 (3)	1.94 (3)	2.808 (4)	159 (3)
N2—H22N···O4	0.90 (3)	2.05 (3)	2.944 (2)	173 (3)
N2—H23N···O16	0.90 (3)	1.90 (3)	2.783 (3)	164 (3)
O3—H3O···O6^ii^	0.66 (3)	1.92 (3)	2.570 (2)	169 (4)
O5—H5O···O2^i^	0.76 (3)	1.84 (3)	2.592 (2)	170 (3)
O8—H8O···O11^iii^	0.70 (3)	1.81 (3)	2.508 (2)	169 (3)
O12—H12O···O9^iv^	0.83 (3)	1.71 (3)	2.517 (2)	165 (3)
O13—H131···O6^ii^	0.79 (3)	1.92 (3)	2.691 (2)	166 (3)
O13—H132···O20A	0.77 (3)	1.92 (3)	2.671 (3)	165 (3)
O14—H141···O18A^v^	0.82 (3)	1.91 (3)	2.697 (3)	163 (3)
O14—H142···O9^iv^	0.74 (3)	1.95 (3)	2.688 (2)	172 (3)
O15—H151···O1	0.80 (3)	2.01 (3)	2.803 (2)	177 (3)
O15—H152···O14	0.66 (3)	2.50 (3)	2.946 (2)	127 (3)
O15—H152···O12^iv^	0.66 (3)	2.54 (3)	3.049 (2)	136 (3)
O16—H161···O15^i^	0.85 (4)	1.94 (4)	2.788 (3)	176 (3)
O16—H162···O17^i^	0.89 (4)	1.97 (4)	2.844 (3)	170 (3)
O17—H171···O21^vi^	0.75 (3)	2.14 (4)	2.795 (3)	147 (3)
O17—H172···O18A^vii^	0.73 (4)	2.13 (4)	2.845 (3)	167 (4)
O19—H191···O13	0.84 (4)	2.27 (4)	3.063 (3)	158 (3)
O19—H192···O2^v^	0.75 (4)	2.04 (4)	2.773 (3)	166 (4)
O21—H211···O19	0.80 (4)	2.05 (4)	2.815 (3)	162 (4)
O21—H212···O19^viii^	1.00 (4)	1.93 (4)	2.914 (3)	169 (3)

Symmetry codes: (i) *x*, −*y*+3/2, *z*−1/2; (ii) −*x*+1, −*y*+1, −*z*+1; (iii) −*x*, *y*+1/2, −*z*+1/2; (iv) −*x*, −*y*+1, −*z*+1; (v) −*x*+1, *y*−1/2, −*z*+3/2; (vi) −*x*+1, *y*+1/2, −*z*+3/2; (vii) −*x*+1, −*y*+2, −*z*+1; (viii) −*x*+1, −*y*+1, −*z*+2.
